# Flattening of Diluted Species Profile via Passive Geometry in a Microfluidic Device

**DOI:** 10.3390/mi10120839

**Published:** 2019-11-30

**Authors:** Michael Miles, Biddut Bhattacharjee, Nakul Sridhar, Apresio Kefin Fajrial, Kerri Ball, Yung Cheng Lee, Michael H. B. Stowell, William M. Old, Xiaoyun Ding

**Affiliations:** 1 Department of Mechanical Engineering, University of Colorado, Boulder, CO 80309-0552, USA; Michael.Miles@colorado.edu (M.M.); Nakul.Sridhar@colorado.edu (N.S.); Apresio.Fajrial@colorado.edu (A.K.F.); y.c.lee@colorado.edu (Y.C.L.); michael.stowell@colorado.edu (M.H.B.S.); 2 Department Molecular, Cellular and Developmental Biology, University of Colorado, Boulder, CO 80309-0552, USA; biddut.bhattacharjee@colorado.edu (B.B.); kerri.ball@colorado.edu (K.B.)

**Keywords:** microfluidics, lab on a chip, flow profile, flow control

## Abstract

In recent years, microfluidic devices have become an important tool for use in lab-on-a-chip processes, including drug screening and delivery, bio-chemical reactions, sample preparation and analysis, chemotaxis, and separations. In many such processes, a flat cross-sectional concentration profile with uniform flow velocity across the channel is desired to achieve controlled and precise solute transport. This is often accommodated by the use of electroosmotic flow, however, it is not an ideal for many applications, particularly biomicrofluidics. Meanwhile, pressure-driven systems generally exhibit a parabolic cross-sectional concentration profile through a channel. We draw inspiration from finite element fluid dynamics simulations to design and fabricate a practical solution to achieving a flat solute concentration profile in a two-dimensional (2D) microfluidic channel. The channel possesses geometric features to passively flatten the solute profile before entering the defined region of interest in the microfluidic channel. An obviously flat solute profile across the channel is demonstrated in both simulation and experiment. This technology readily lends itself to many microfluidic applications which require controlled solute transport in pressure driven systems.

## 1. Introduction

In recent years, microfluidic devices have become an important tool for use in lab-on-a-chip (LoC) processes, including drug screening and delivery, biochemical reactions, sample preparation and analysis, separation, chemotaxis, and cell manipulation [1,2,3,4,5,6]. Compared with traditional methods, these devices have the potential to simplify laboratory processes while reducing the time and cost required. Generally, fluids containing a sample are driven through a device consisting of microfabricated channels that complete the required experimental steps. To transport the fluid, either a pressure gradient or electroosmotic force is commonly used [7,8,9,10].

In order to maintain precise solute transport inside the microchannel, which is paramount for molecular- or cellular-level application, a flat cross-sectional concentration profile is desired [11]. For example, the flat concentration profile has been demonstrated to improve biochemical assay technology by allowing a uniform fluid velocity profile to disperse an equal amount of sample to be analyzed in parallel [12]. The most common technique to achieve a flat concentration profile is by applying electrical potential to drive the liquid motion, which is popularly described as electroosmotic flow (EOF). The implementation of EOF inherently produces a nearly uniform velocity profile across a channel [13,14,15]. However, EOF heavily relies on the electrical properties of the medium and surface, for instance, imperfections or chemical coatings on the channel surface can distort the concentration profile [16,17]. Moreover, the application of an electric field can potentially be incompatible with nonhomogeneous solutes/buffers used in the device and may have negative effects on biological samples [18,19]. It can also be difficult to use electroosmotic flows for particular processes, such as mixing particles, due to the irrotational nature of the flow [20,21]. Recently, flat concentration profiles could be generated through magnetohydrodynamic (MHD) pumping [22]. To allow for effective MHD, the introduction of redox species to the fluid element is necessary. Thus, controlling the concentration profile in a microfluidics device without dependency on the liquid composition remains a challenge.

Pressure-driven flows introduced with a mechanical syringe pump provide a simple and inexpensive alternative when EOF is not compatible with a device or the sample [13,23]. However, it is well known that a pressure gradient through a channel will introduce a parabolic flow profile in the axial direction. This is due to the no-slip condition found at the channel wall, which causes a non-uniform velocity gradient along the cross section of the channel [24]. Considering a two-dimensional (2D) cross-section of a rectangular channel, this effect is proportional to channel dimensions and much more significant in the axial dimension of a typical device. Accordingly, the introduction of such a parabolic profile prevents precise solute transport within the device.

Related literature on the topic of Taylor dispersion in microfluidic channels has suggested that passive geometric features can be utilized to control the solute profile. Dutta et al. showed in theory and simulation that the length of a solute slug undergoing Taylor dispersion can be significantly reduced in rectangular and isotropically wet-etched microfluidic channels by increasing the cross-sectional area near channel side walls [25]. This reduces the effective drag of the walls on the fluid as a result of no-slip conditions. Dutta et al. expounded on this work to include elliptical and trapezoidal channel geometries [26]. Despite significant theoretical contributions, these works lack experimental proceedings to test the developed theories and simulations. Similarly, Aminian et al. reported with theoretical and experimental evidence that cross-sectional aspect ratio alone could control the shape of the concentration profile of a solute plug in the axial direction [27], however, they primarily concerned themselves with long-term flow behavior, which is not directly applicable to many LoC processes requiring well-behaved solute distribution within a short timescale of injection. Recently, Gökçe et al. reported self-coalescence-based control of reagent reconstitution by implementing a special geometric feature—capillary pinning line—into the shallow microfluidic channel [28]. Although reagent delivery was effectively demonstrated, the design is limited by the fact that dried reagents need to be pre-positioned at specific locations of the device, and the delivery area transverse dimension (1.5 mm) is quite small.

Here, we introduce an elegantly simple method for achieving a flat solute concentration profile in a microfluidic channel, solely relying on the modified geometry of merely the entry segment of the channel. We start with a typical microchannel found in LoC applications (hereto referred to as the “Control” channel design) and draw inspiration from finite element fluid dynamics simulations to design and fabricate passive geometric features within the diverging entry segment to achieve a flat solute concentration profile (hereto referred to as the “Curve” channel design). We fabricate the device by utilizing conventional machining technology due to its complex shape. For analyzing the device performance, we concern ourselves with achieving a flat solute profile across the channel width (y). Due to the 2D nature of the microchannel, a flat solute profile across the channel depth (z) is not considered. Performance of the features, as characterized by the transformation of an inherent parabolic solute profile into a flat solute profile, is measured experimentally and compared with simulation results.

## 2. Theoretical Methods

### 2.1. Theory

**Figure 1:**
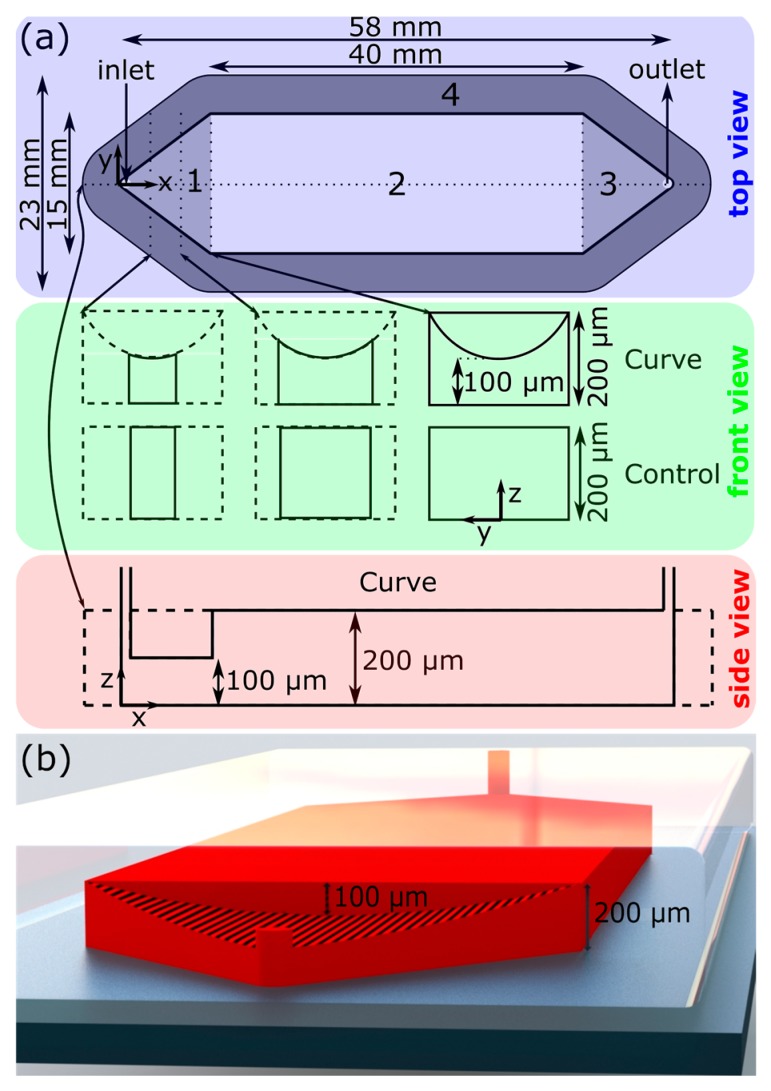
(**a**) Schematic of the microfluidic device—it shows dimensions and labels for the four primary regions: (**1**) Upstream region with varying depth (see the front view); (**2**) Midstream region with uniform cross-section (see the side view); (**3**) Downstream region; (**4**) Bonding region. (**b**) CAD rendering of the Curve microfluidic device. PDMS (Polydimethylsiloxane) = transparent; fluid domain = red; glass slide = grey. Scaled and labelled for illustrative purposes.

In a microfluidic channel as shown in Figure 1a, the straight segment in the middle of the channel is connected to either inlet or outlet through a diverging triangle segment. The aspect ratio, that is, depth to width, of the straight segment is 0.0133, and the depth is constant over this segment. Hence, the horizontal velocity profile does not change significantly, except near the side walls of the channel. In the Control design, the depth of the channel is constant everywhere in both diverging and straight segments, the depth-averaged constant pressure lines can be approximated by concentric arcs. The fluid velocity field is dominated by the radial velocity, where the center is located at the intersection of the two vertical walls of the diverging segment. When a solute slug is introduced at the inlet, the horizontal profile—viewed from the top—is predominantly circular. This circular profile enters the straight segment of the channel. 

To start with a nearly straight-line horizontal profile of the second liquid at the joining region of the diverging and straight segments of the channel, the proposed design has a modified cross-sectional area in the diverging segment. Here, the roof of the channel is such that the depth varies as a parabola where the apex—shallowest point of the channel—coincides with the line between the inlet and the outlet. To gain some insight about how this design contributes toward correcting the circular horizontal profile of the second liquid, let any point on the top surface of the channel be described by *z = y^2^*, where the origin is located at the intersection of the two side walls on the bottom surface of the channel, *z* is the vertical position, *y* is the position along the breadth, and *x* is the axial position. In addition, we consider a cylindrical coordinate system (*r,θ,z*) with the same origin, where *θ* is the angular position with respect to the straight line between the centers of the inlet and outlet. For any given *θ*, we find ∂*z*/∂*r* = 2*r*∙sin^2^*θ*, and for any given radial position, *r*: ∂*z*/∂*θ = r*^2^∙sin2*θ*. Therefore, the depth increases linearly with the distance from the origin at any given angular position. This effect is stronger with higher values of *θ*, toward the side walls, due to the multiplying factor sin^2^*θ*. The second equation implies that for a given radial position, depth increases nonlinearly as sin2*θ*. Again, this effect is intensified by the factor *r*^2^, signifying that further from the inlet, the increase in depth is significant as we move near the side walls. Thus, we can qualitatively assess the contribution of the modified diverging segment to the correction of velocity field. The depth-averaged pressure at any given radial position will vary significantly with angular position. Specifically, the pressure along the central axial line will be minimal, and will increase significantly near the side walls. Consequently, there will be a significant tangential component of velocity (*u_θ_*) in addition to the radial component (*u_r_*). The horizontal front/profile of the second liquid will no longer appear circular due to increased flow near the side walls. Overall, the front of the second liquid will be straightened as it enters the main uniform segment of the channel.

### 2.2. Simulation

Using the theoretical intuition discussed above, several geometric modifications to the upstream region of the Control channel were considered to yield the flat concentration profile. Various Curve channel designs were drafted with the intent of increasing cross-sectional area near the side walls of the upstream region of the channel relative to the cross-sectional area in the center of the channel (y = 0). In turn, fluid velocity near the edges will increase such that the solute profile near the edges will “catch up” to the profile in the center. Taylor’s hypothesis suggests that extension of the geometric features to the entirety of the channel is not necessary on the relevant time and length scales. Moreover, such features further complicate manufacturing and may cause unpredictable biases in applications which require cell culture or other operations in which oxygen permeability of the polydimethylsiloxane (PDMS)-based microchannel is a factor.

The Curve channel designs under consideration were modelled in SolidWorks and simulated in COMSOL Multiphysics (COMSOL Inc., Stockholm, Sweden). Simulations were performed with volumetric flow rates of 115, 230, 460, and 920 µL/min, a relevant range for LoC applications. In all models, the inlets to the channel were modelled as a planar face perpendicular to the flow ($\hat{n}=-x$) and coincident with the central axis of the inlet. COMSOL simulations made use of the Laminar Flow and Transport of a Diluted Species physics modules. The velocity field from the laminar flow module was used for the transport properties of the diluted species module. Studies were performed in a two-step process: Step 1 was a Stationary step, which was used to simulate the developed laminar flow at steady state, and Step 2 was a Time-Dependent step, which was used to simulate the species transport in the steady-state flow with respect to time. COMSOL simulation parameters are listed in Table S1 in the supplementary materials. 

### 2.3. Channel Design

Ultimately, we chose a design resembling a typical microchannel with an extruded parabolic mass in the upper region. A schematic of the channel design is presented in Figure 1. The design was selected due to its superior performance in simulation and constitutes the “Curve” channel for the remainder of the study. The design effectively increases the cross-sectional area near the side walls, as discussed above, by squeezing the fluid near the center of the channel. Under the range of flow rates and timescales, the simulated solute profile is extremely flat with respect to the y–z plane. Furthermore, despite the complex shape of the channel in the context of microfabrication, it was one of the simpler designs under consideration in terms of fabrication.

As mentioned, the Curve channel is best described as a typical microchannel (“Control”) with minor geometric modifications only to the upstream region (region 1 in Figure 1a). Each channel features one inlet and one outlet, both diameter 1 mm, separated by a straight channel. The midstream rectangular region (Region 2 in Figure 1a) is uniform with a cross-section of 15 mm by 40 mm, and is the primary space in which the LoC operations take place. The upstream and downstream 9.7 mm long sections of the channel (1 and 3 in Figure 1, respectively) are triangular: the upstream triangular region is purposed to expand the fluid from the inlet to the midstream straight region, and the downstream triangular region contracts the fluid from the midstream region to the outlet. The Control channel was 200 µm tall everywhere. Due to manufacturing difficulties discussed in Section 4.2, the Curve channel was 225 µm tall throughout the majority of Region 2 (Figure S1 in supplementary materials). The geometric modifications which constitute the Curve design can be described by a parabolic curve drawn at the upstream–midstream intersectional plane (x = 0), with endpoints at the corners of the ceiling–side wall intersectional edges ((y,z) = (±7.5 mm, 200 µm)) and a depth of 100 µm (z = 100 µm). This curve is then extruded in the −x direction up to and beyond the inlet of the channel.

## 3. Experimental Methods

### 3.1. Channel Fabrication

Since the Curve channel design requires a smooth, multidimensional curved surface, traditional microfabrication techniques such as photolithography or deep reactive-ion etching (DRIE) were not an option. Initially, we experimented with fabricating via high-resolution three-dimensional (3D) printing (Stratasys Objet Pro, vertical resolution 16 µm), as this new technology very readily lends itself to this difficult fabrication problem. We followed the protocol outlined in King’s research to enable curing of PDMS in contact with the cured objet material, but this resulted in thermal warping and inconsistent performance results [29].

The final Curve channel was fabricated from a solid aluminum block via computer numerical control machining. Prior to machining, the aluminum stock was finely lapped and polished. This polished face is planar with the “ceiling” of the final channel mold in the midstream and downstream regions, in an effort to minimize artifacts of the machining process. The upstream region of the mold was machined with only flow-parallel (x-parallel) tool paths so to reduce asymmetric species transport in the region. Sylgard 184 (Dow Corning, Midland, MI, USA) PDMS was prepared by mixing the monomer with the curing agent in a 10:1 mass ratio and poured over the respective channel mold. The PDMS was allowed to cure at 70 °C for 1 h. After curing, the PDMS was carefully peeled from the mold, inlet/outlet holes were punched, and the PDMS was bonded to a standard glass microscope slide via oxygen–plasma bonding. A glass microscope slide was also cut to length and bonded on top to prevent deflection of the PDMS under pressure, however, this is not depicted in the figures presented for visualization purpose.

### 3.2. Optical Concentration Measurement

**Figure 2:**
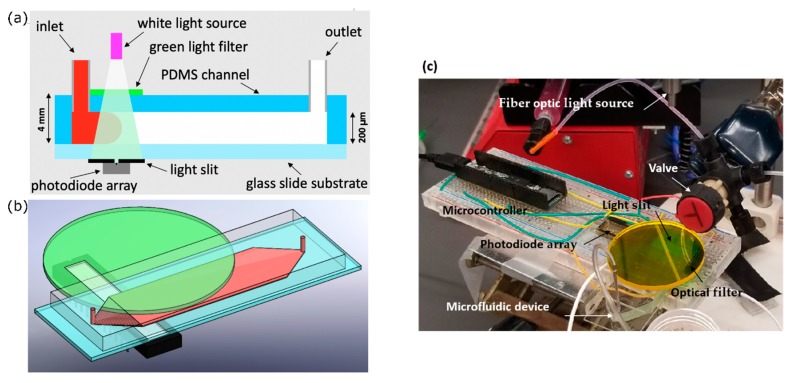
(**a**) Schematic and (**b**) CAD rendering of the optical concentration measurement system. (**c**) Experimental setup. Photodiode array is right under the channel to detect the light from the top to measure phenol red concentration in upstream or downstream of the channel.

An optical absorption system was used to experimentally assess Curve channel performance. The setup for this optical absorption system is depicted in Figure 2. Phenol red, a pH-sensitive red dye, was prepared in 40 µM DI (deionized water) water solution and adjusted to pH = 7.2. At this pH, phenol red exhibits peak absorption at 560 nm, which falls in the visible green spectrum. 

First, white light was passed through a green light filter. The resulting green light passed through the channel, and then through a light slit to block reflected light, and finally into a one-dimensional (1D) linear photodiode array (TAOS TSL1406, pixel resolution 63.5 µm, ams AG, Premstaetten, Austria) placed parallel to the y-direction, so as to capture the transmitted light intensity (z-averaged concentration) across the entire width of the channel. Next, the microchannel was flushed with DI water, and then a slug of phenol red solution was introduced into the inlet via syringe pump. The outlet was left to drain freely. Incident light intensities were read by the photodiode array every t_PD_ = 50 ms by a Teensy 3.6 with custom Teensy-duino code (an Arduino wrapper for the Teensy, Teensy 3.6). A “pseudo-snapshot” of the concentration profile was then derived by measuring concentration across the width of the channel at some longitudinal x position over time. These measurements are generated into a single image by calculating linear fluid velocity from the channel dimensions and volumetric flow rate. This is justified by an alternative perspective on Taylor’s “frozen turbulence” Hypothesis—an observation of a concentration slug in laminar flow at a single point will agree well with the actual progression of the concentration slug in time. Furthermore, while the concentration profile gap does noticeably widen with respect to flow rate, it does not do so with respect to time, thus providing a sufficient means of capturing the concentration distribution in the whole channel at a given point in time. 

In order to assess performance, incident light measurements were recorded from the initial presence of the DI water until the phenol red slug had fully saturated the channel. The first and last few measurements were discarded to prevent anomalies resulting from transient fluid flow. Measurements from pixels outside the boundaries of the channel were also discarded. Of the remaining data, for each pixel, measurements were normalized by the first measurement, which corresponds to the pixel value for DI water.

## 4. Results and Discussion

### 4.1. Simulation

**Figure 3:**
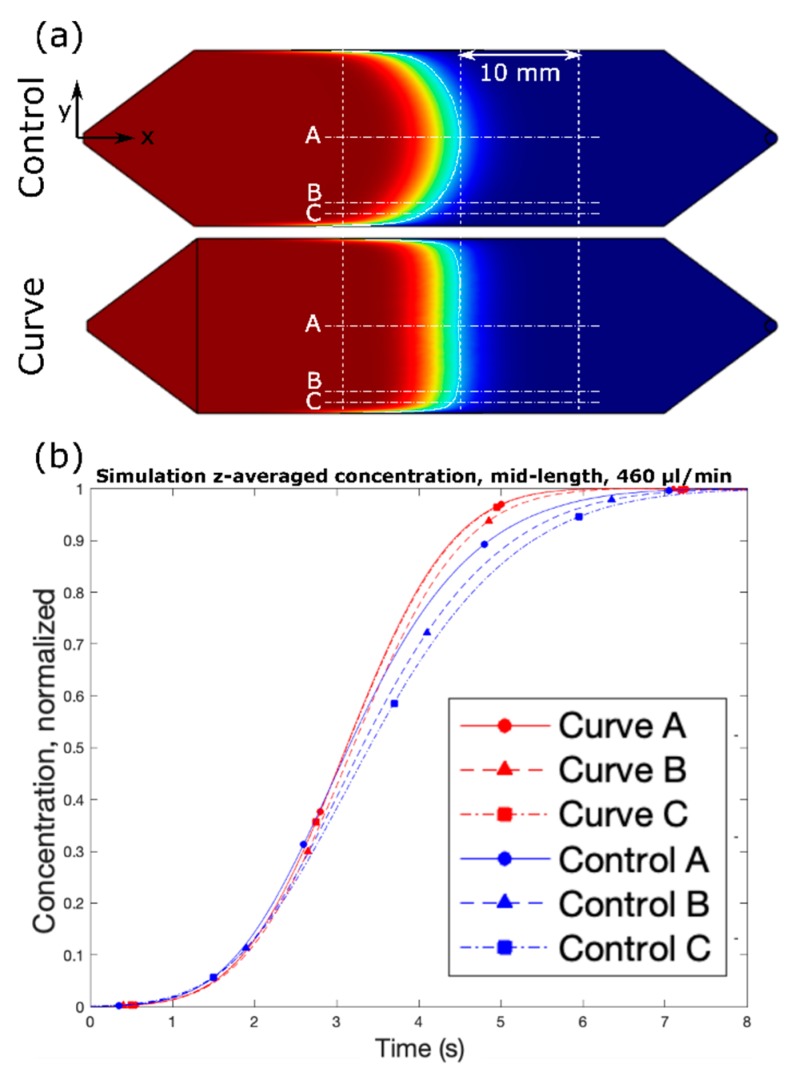
(**a**) COMSOL Multiphysics simulations of Control (**top**) and Curve (**bottom**) channel designs, and (**b**) simulated z-averaged normalized concentration over time for both designs. Q = 460 µL/min.

The COMSOL simulation showed that the Curve channel is able to create a flat concentration profile spanning more than 95% of the width of the channel, as can be seen in Figure 3a. We characterized the flattening profile by simulating and comparing the solute concentration at different distances along the channel width, which are at points A, B, and C. Point A is located in the middle of the channel (7.5 mm from the bottom edge). Points B and C are measured at 2 mm and 1 mm from the bottom edge of the channel, respectively.

**Figure 4:**
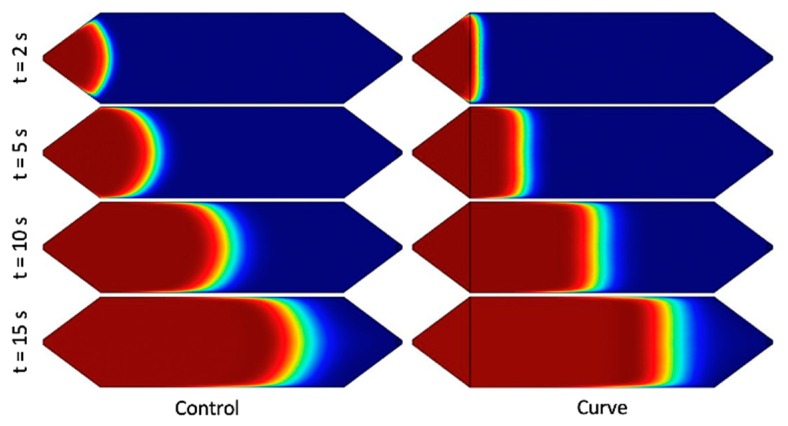
COMSOL Multiphysics simulations of Control (**left**) and Curve (**right**) channel designs at various time points. Q = 460 µL/min, z = 100 µm (mid-depth).

Parabolic flow profile usually has the time lag in the dispersion of the solute concentration at the different y-axis location. This typical condition for the Control channel can be easily identified by comparing those aforementioned points. Figure 3b shows the normalized z-average solute concentration as measured along points A, B, and C from the lengthwise (x-axis) midpoint of the channel. To achieve 0.95 concentration of the dispersed solute in the control channel at points A, B, and C, it takes 5.3125 s, 5.6312 s, and 5.9187 s, respectively. There is a clear delay up to 10% (0.6 s) of the time length to disperse the same amount of solute between the leading point and the edge point. The flattening effect of the Curve channel is clear—the delay between points A and C in the Control channel is about 500 ms, with point B falling roughly in the middle; meanwhile, the delay between all points, A, B, and C, in the Curve channel is a fraction of the time, and nearly indistinguishable. Moreover, the flattening effect of the Curve channel occurs immediately, since the beginning of fluid profile development. In Figure 4, we present the flow profile at different x-axis locations, which represent the amount of time required to disperse the solute at those positions. The flat fluid profile in the Curve channel is achieved directly after progressing from the modified upstream region. These simulations were also performed at various flow rates between 115 and 960 µL/min (Figure S2 in supplementary materials), and all showed similar trends, with the only noticeable effect being a positive correlation between increased flow rate and the concentration profile gap width due to Taylor dispersion.

### 4.2. Experiment

**Figure 5:**
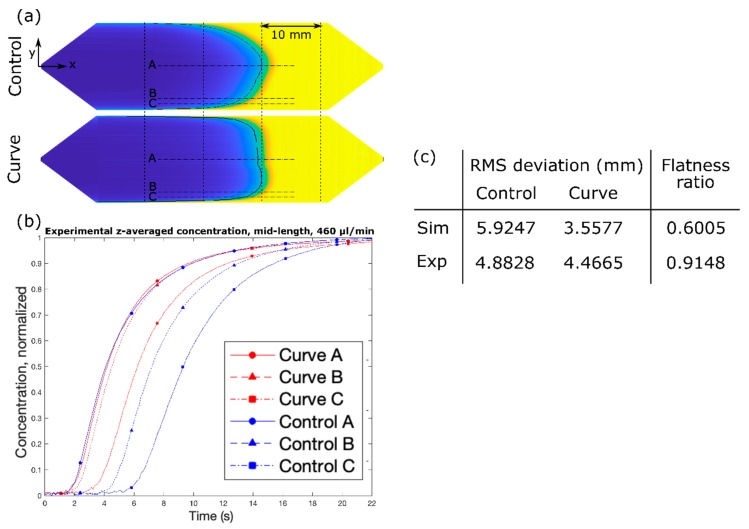
(**a**) Flow experiments of Control (**top**) and Curve (**bottom**) channel designs, and (**b**) experimental z-averaged normalized concentration over time for both designs. (**c**) root-mean-square (RMS) deviation and flatness ratio for simulated and experimental data. Q = 460 µL/min.

Using the design that we have confirmed by simulation, we sought to experimentally demonstrate significant flattening of the concentration profile. Figure 5 shows the optical measurement data of Phenol red dye using the fabricated channels and the setup in Figure 2. Similar to the case of the simulated channels, though to a slightly lesser extent, significant flattening of the concentration profile has occurred. While the time delays between the center and edge points are quite longer in the experimental results, the trend observed in Figure 3 still holds true. In the case of the Control channel, the delay between points A and C is about 6 s, with point B falling roughly in the middle; meanwhile, in the case of the Curve channel, the delay between points A and C is about 2 s, and point B lags point A by only about 500 ms. 

We assessed “flatness” by calculating the root-mean-square (RMS) deviation between the straight line tangent to the leading point of the slug and a contour fit to the concentration profile at 50% solute concentration. The ratio between the RMS deviation as calculated for the curve design to that for the control design provides a simple, bounded metric for assessing the improvement in the flatness of the profile due to the introduction of the passive geometric feature, in which a flatness ratio of 1 indicates no effect of the feature and a flatness ratio of 0 indicates a perfectly flat concentration profile. It is noted that a ratio of 0 is impossible to achieve in a pressure-driven flow of real fluids due to the boundary layer effect of the two vertical walls. Using this metric, we calculated RMS deviation of 4.4665 mm and 4.8828 mm for the proposed Curve and Control designs, respectively, resulting in a flatness ratio of 60% in simulation (Figure 5c). This ratio is the limiting value achievable under a best case scenario, that is, with a perfect device but identical material and flow conditions. However, the flatness ratio of 91% was achieved in the experiment. As seen in Figure 5a, the profile length for the Control channel is nearly double that of the Curve channel. A flow experiment with black dye was also carried out to directly visualize the flow front profile, which matches well to the simulation results in Figure 4, as shown in Figure S3 in supplementary materials.

While significant flattening of the concentration profile was achieved, we recognize it displays a performance inferior to that suggested by the simulation results for a channel of the same geometry. There are several reasons for this. The primary reason is that, as measured by stylus profilometry (Figure S1 in supplementary materials), the Curve channel mold exhibited notable errors and artifacts of CNC machining that highlight the difficulty in using traditional manufacturing processes on devices of such small size. As mentioned in Section 2.3, despite being designed with a height of 200 µm, the Curve channel was manufactured with a height of about 225 µm due to resolution constraints. Further, the geometric feature cut into the mold was very rough and moderately asymmetric. Perhaps most detrimental, due to limitations in spatial resolution compared with traditional microfabrication procedures, the sharp edges that make up the steeper portions of the parabolic curve cannot be manufactured sufficiently fine, resulting in a concentration profile that lacks definition at its edges as well. However, we believe the proposed design can be realized with better dimensional accuracy through the high-precision (~1 µm) CNC machining of the mold, a better 3D printer with layer resolution less than 10 µm, or an advanced microfabrication technique such as photoresist reflow process [30]. Additionally, it is likely that we used imperfect model settings in our simulations that resulted in slightly different fluid flow behavior than expected, however, the general trend of the fluid behavior still agrees well with the experimental results.

Despite the aforementioned fabrication challenges, we have exhibited significant solute concentration profile flattening via passive geometry. Further, this achievement has been performed in a practical manner with a microchannel design typical to that found in most LoC applications. Thus, this simple and general technology readily lends itself to LoC applications which require controlled solute transport in pressure driven systems.

## Supplementary Materials

The following are available online at https://www.mdpi.com/2072-666X/10/12/839/s1, Table S1: COMSOL Multiphysics simulation settings, Figure S1: Stylus profilometry (Dektak XT) characterization of CNC’s aluminum mold for Control (**left**) and Curve (**right**) channels at Region 1–2 interface (x = ~9 mm), Figure S2: COMSOL Multiphysics simulations of Control (**left**) and Curve (**right**) channel designs at various flow rates of interest in many microfluidic applications, Figure S3: Flow experiment with black dye for visualization purposes in control channel and curve channel at multiple time points.
