# Supplementary material for: Flattening of Diluted Species Profile via Passive Geometry in a Microfluidic Device
Source: Micromachines (Basel). 2019 Nov 30;10(12):839. doi: 10.3390/mi10120839 (PMC6952922; doi:10.3390/mi10120839)
Supplement: Supplementary file 1 [file micromachines-10-00839-s001.pdf]

Supplementary Materials: Flattening of Diluted Species Profile via Passive Geometry in a Microfluidic Device

Michael Miles [1], Biddut Bhattacharjee [2], Nakul Sridhar [1], Apresio Kefin Fajrial [1], Kerri Ball [2], Yung Cheng Lee [1], Michael H. B. Stowell [1,2], William M. Old [2,*] and Xiaoyun Ding [1,*]

[1] Department of Mechanical Engineering, University of Colorado, Boulder, CO 80309-0552, USA; Michael.Miles@colorado.edu (M.M.); Nakul.Sridhar@colorado.edu (N.S.); Apresio.Fajrial@colorado.edu (A.K.F.); y.c.lee@colorado.edu *(Y.C.L.);* michael.stowell@colorado.edu (M.H.B.S.)

[2] Department Molecular, Cellular and Developmental Biology, University of Colorado, Boulder, CO 80309-0552, USA; biddut.bhattacharjee@colorado.edu (B.B.); kerri.ball@colorado.edu (K.B.)

* Correspondences: William.Old@Colorado.edu (W.M.O.); Xiaoyun.Ding@Colorado.edu (X.D.)

1. Method for Assessing Flatness

We assessed "flatness" by calculating the root-mean-square (RMS) deviation between the solute concentration profile and its leading edge. Using this measure, a solute slug with a perfectly straight and y-parallel concentration profile would have an RMS deviation of 0. A parabolic concentration profile with a very long tail can theoretically have a deviation up to infinity. The ratio of the Curve RMS deviation to the Control RMS deviation provides a simple, bounded metric for assessing the improvement in the flatness of the concentration profile due to the introduction of the passive geometric feature. A flatness ratio of 1 indicates zero effect of the feature, and a flatness ratio of 0 indicates ideal flattening of the profile by the feature.

In the case of the Simulation data, the solute profile was defined as the 50% concentration isoline in the xy plane (z = 100 μm) and exported from COMSOL. From this, the RMS deviations between the isoline and its leading edge for both the Curve and the Control, and the resultant "flatness" ratio, were calculated.

In the case of the Experimental data, some image processing was first necessary. To start, the edges of the channel were manually identified from the experimentally collected "pseudo-snapshot" of the concentration profile. Next, all the measurements (along the x axis) from each pixel (aka photodiode element) were normalized by the first measurement from that pixel to resolve inter-pixel discrepancies. Finally, as in the case of the Simulation data, the 50% concentration isoline was extracted from the measurements, and the RMS deviations for each design and their ratio were calculated.

There is a significant lag between the Simulation Control profile and the Experimental Control profile for two primary reasons. First, while the Simulation data was generated with an ideal source at the base of the channel, the solute slug captured in the Experimental data travelled through some minimal plumbing before entering the channel, lengthening its profile in the x (flow-parallel) direction. Second, the Simulation data was captured precisely in the xy plane at z = 100 μm; meanwhile, the Experimental data was z-averaged, so the presence of the vertical (xz parallel) parabolic profile in the channel degraded the crispness of the measurements.

2. Supplementary Tables and Figures

Table S1. COMSOL Multiphysics simulation settings.

Items	Parameters
COMSOL Simulation Parameters	Material: Water, liquid<br>Laminar flow
General	Compressibility: Incompressible flow<br>Turbulence model type: None<br>Neglect inertial term <unchecked><br>Enable porous media domains <unchecked><br>Reference pressure level: 1 atm
Fluid Properties	Temperature: 293.15 K<br>Density: from material<br>Dynamic viscosity: from material
Initial Values	Velocity field: [0, 0, 0] m/s<br>Pressure: 0 Pa
Wall	Boundary conditions: No slip
Inlet	Boundary condition: Mass flow<br>Mass flow type: Mass flow rate<br>Normal mass flow rate: 460 uL/min
Outlet	Boundary condition: Pressure<br>Pressure: 0 Pa<br>Normal flow: <unchecked><br>Suppress backflow: <checked>
Transport of Diluted Species	
General	Convection: <checked><br>Migration in electric field: <unchecked><br>Adsorption in porous media: <unchecked><br>Dispersion in porous media: <unchecked><br>Volatilization in partially saturated porous media: <unchecked>
Transport Properties	Velocity field: Velocity field (spf)<br>Diffusion material: from material<br>Diffusion coefficient: 5.16×10^{-10} m^2/s, isotropic
Initial Values	Concentration: 0 mol/m^3
Inflow	Concentration: 0.04 mol/m^3<br>Boundary condition type: Concentration
constraint	--

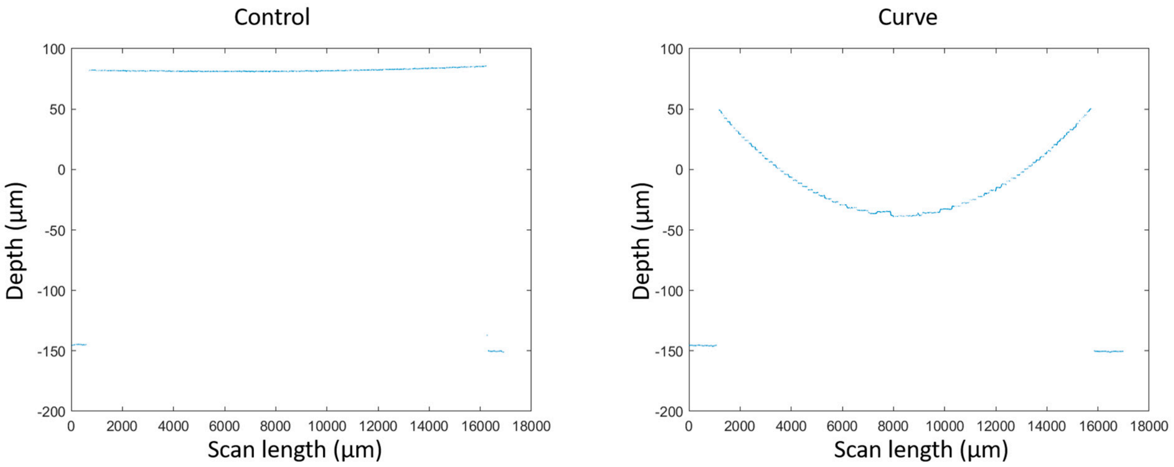

Figure S1. Stylus profilometry (Dektak XT) characterization of CNC's aluminum mold for Control (**left**) and Curve (**right**) channels at Region 1–2 interface (x = ~9 mm).

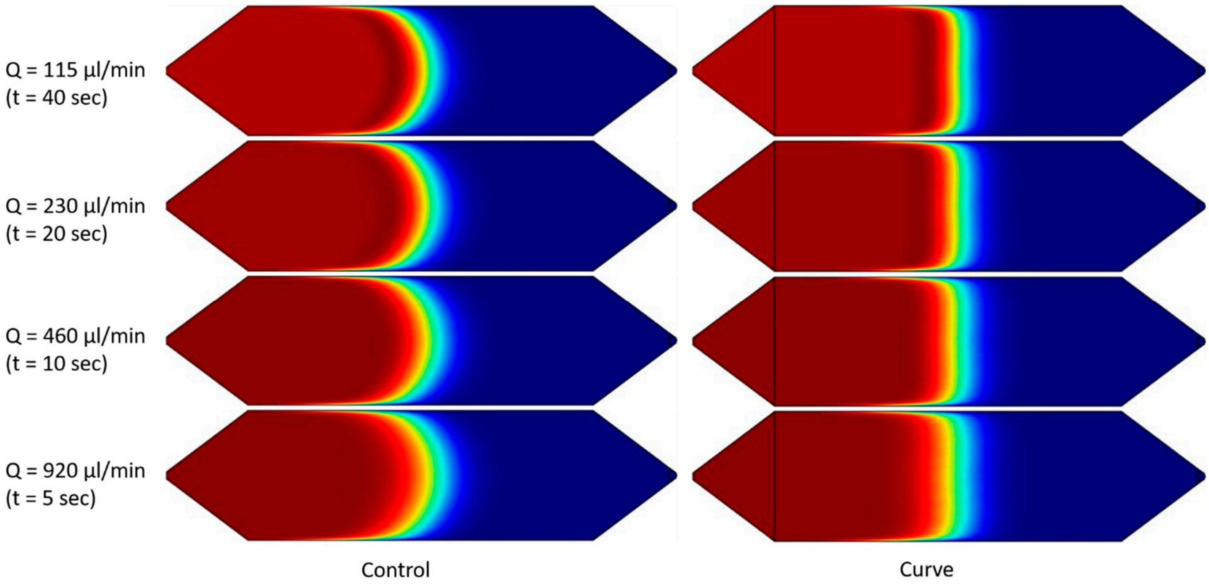

Figure S2. COMSOL Multiphysics simulations of Control (**left**) and Curve (**right**) channel designs at various flow rates of interest in many microfluidic applications. Time points were selected such that the concentration gradient was at channel mid-length, z = 100 µm (mid-depth). Note, the larger dispersion gaps in the cases of higher flow rate.

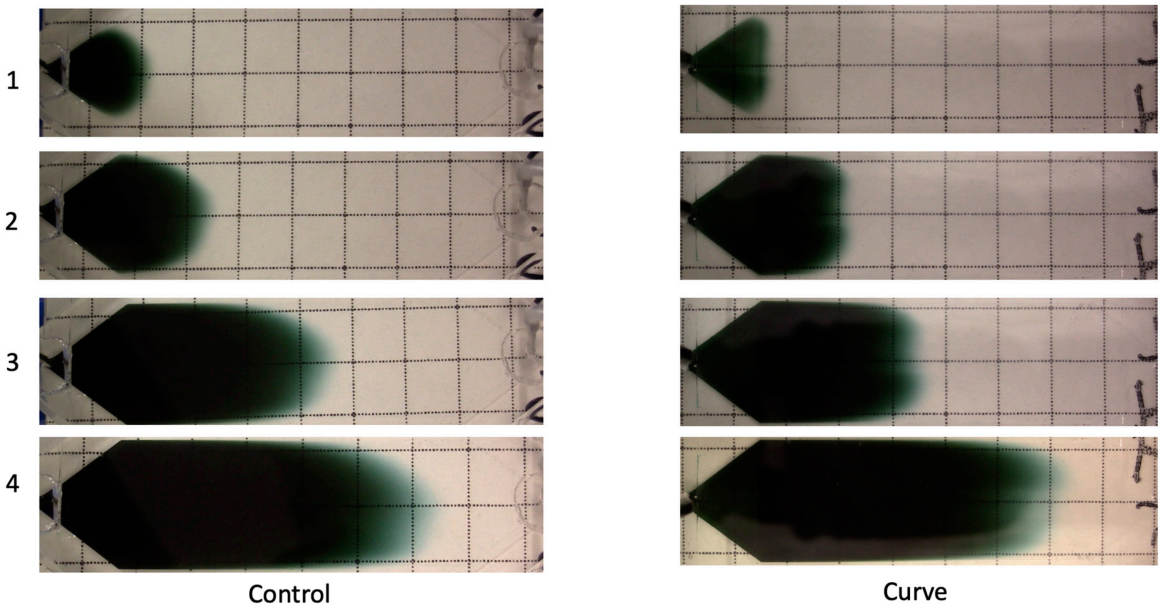

Figure S3. Flow experiment with black dye for visualization purposes in control channel (**left**) and curve channel (**right**) at multiple time points.
